# Comparative effectiveness of the BNT162b2 and ChAdOx1 vaccines against Covid-19 in people over 50

**DOI:** 10.1038/s41467-022-29159-x

**Published:** 2022-03-21

**Authors:** Junqing Xie, Shuo Feng, Xintong Li, Ester Gea-Mallorquí, Albert Prats-Uribe, Dani Prieto-Alhambra

**Affiliations:** 1 Centre for Statistics in Medicine, NDORMS, University of Oxford, Oxford, UK; 2 Oxford Vaccine Group, Department of Paediatrics, University of Oxford, Oxford, UK; 3 Nuffield Department of Clinical Medicine, University of Oxford, Oxford, UK

**Keywords:** Viral infection, SARS-CoV-2, Vaccines, Epidemiology

## Abstract

Although pivotal trials with varying populations and study methods suggest higher efficacy for mRNA than adenoviral Covid-19 vaccines, not many studies have directly compared vaccine effectiveness in the population. Here, we conduct a head-to-head comparison of BNT162b2 versus ChAdOx1 against Covid-19. We analyse 235,181 UK Biobank participants aged 50 years or older and vaccinated with one or two doses of BNT162b2 or ChAdOx1. People are followed from the vaccination date until 18/10/2021. Inverse probability weighting is used to minimise confounding and the Cox models to derive hazard ratio. We find that, compared with one dose of ChAdOx1, vaccination with BNT162b2 is associated with a 28% (95% CI, 12-42) decreased risk of SARS-CoV-2 infection. Also, two doses of BNT162b2 vs ChAdOx1 confers 30% (95% CI, 25-35) and 29% (95% CI, 10-45) lower risks of both infection and hospitalisation during the study period when the Delta variant is dominant. Furthermore, the comparative protection against the infection persists for at least six months among the fully vaccinated, suggesting no differential waning between the two vaccines. These findings can inform evidence-based Covid-19 vaccination campaigns and booster strategies.

## Abstract (web summary)

Several vaccines have been approved for Covid-19 and effectiveness data could guide public health decisions on vaccine usage. Here, the authors compare effectiveness of BNT162b2 and ChAdOx1 vaccines in people over 50 in the UK and find that BNT162b2 confers lower risk of infection and hospitalization.

## Introduction

To date, four vaccines against the Coronavirus disease 2019 (Covid-19) have been approved for use in the UK by the Medicines and Healthcare products Regulatory Agency: the Pfizer-BioNTech BNT162b2, Moderna’s mRNA-1273, Oxford-AstraZeneca’s ChAdOx1, and Janssen’s Ad26.CoV2.S. Although phase 3 trials suggested that all four have high clinical efficacy, mRNA vaccines demonstrated numerically greater efficacy than adenoviral-based ones: BNT162b2 reported 95% efficacy against symptomatic Covid-19^1^, mRNA-1273 94.1% efficacy^2^, whilst ChAdOx1 had 70.4%^3^, and Ad26.CoV2.S had 66.9% efficacy^4^. However, notable differences in study designs made it difficult to compare vaccine efficacy based on these trials, including different populations recruited in different regions and at different times, diverse primary endpoint definitions, and heterogeneous statistical analysis methods.

Specific for the two more widely utilized vaccines, BNT162b2 and ChAdOx1, several observational studies have recently evaluated their effectiveness in multiple real-world settings from Israel^5^, the UK^6^, and Spain^7^, amongst others. Although useful, none of these studies conducted head-to-head comparisons of vaccine effectiveness. With the ongoing pandemic and rapid rollout of the Covid-19 vaccination programme all over the world, evidence on their comparative performance has become more crucial to inform policy decisions on optimizing vaccine implementation strategies, not only in countries where greater coverage of the prime dose is urgently required or in countries where boost doses and sequential immunization are being considered. Therefore, this study aimed to estimate the comparative effectiveness of BNT162b2 vs ChAdOx1 vaccines against Covid-19 infection and hospitalization in a large and population-based prospective cohort of people aged 50 years or older.

## Results

### Data linkage and study cohorts

**Fig. 1:**
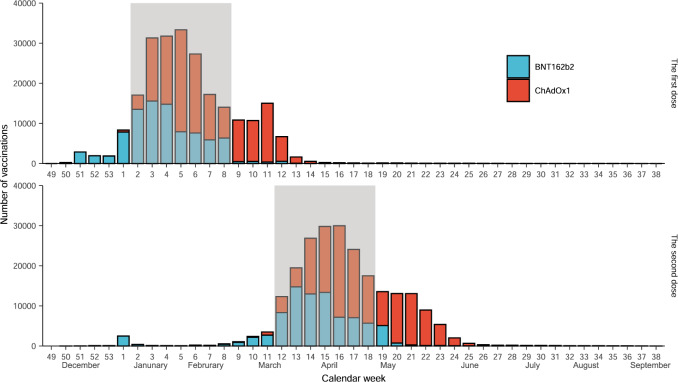
The number of people receiving either BNT162b2 or ChAdOx1 Covid-19 vaccine in the study cohort from Dec 01, 2020 to Sep 21, 2021. The rectangle background with grey colour depicts the vaccination anchor windows. Only people who received study vaccines within the anchor windows were included for the comparative analysis. We defined the 2rd to 8th (Jan 11, 2021 to Feb 28, 2021) and 12th to 18th (March 22, 2021 to May 9, 2021) calendar weeks as the two anchor windows for the one-dose and two-dose cohorts, respectively. The decision-making for these anchor windows was based on (1) there were both BNT162b2 and ChAdOx1 vaccines delivered in each epidemiological week of the window, (2) numbers of the two vaccines were generally comparable, and (3) UK’s policy on the gap between the first and second dose was 10 to 12 weeks for both vaccines.

During the one-dose enrolment period, 70,097 and 98,551 people received the first BNT162b2 and ChAdOx1 Covid-19 vaccines, respectively. These figures were 67,813 and 89,030 accordingly for people receiving the second dose during the two-dose enrolment period. Vaccine uptake over calendar time in our study population is depicted in Fig. 1.

### Baseline characteristics

**Fig. 2:**
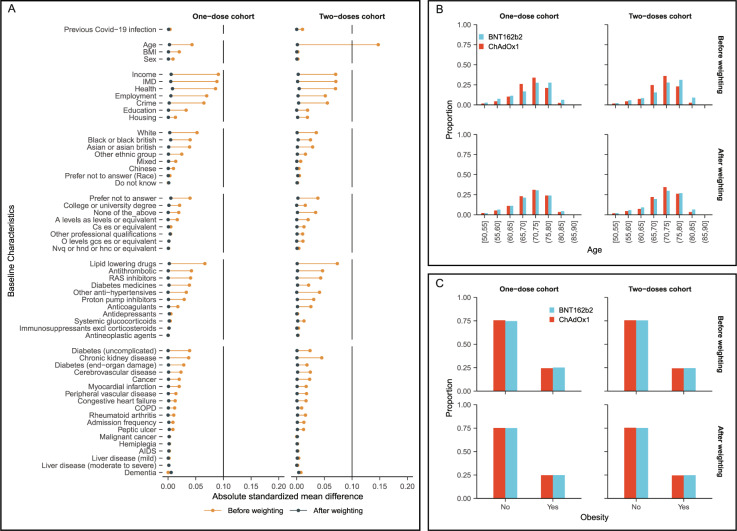
Balance of baseline covariates for the one dose and two doses cohorts, before and after weighting. **A** The standardized mean difference for all covariates included in the propensity score. **B**, **C** The proportion of age categories and obesity by vaccine types.

In the one-dose vaccine cohorts, people receiving BNT162b2 were slightly older (mean (sd) age: 71.35 (7.21) years) than those receiving ChAdOx1 (mean (sd) age: 71.06 (6.02) years). Sex (44.5% vs 44.1% male) and ethnicity (91.2% vs 92.6% White) were comparable between the two groups. Little difference was seen in the prevalence of medicines or comorbidities. The main differences between cohorts were vaccination dates and socio-economic factors such as income (Fig. 2 and Supplementary Table 2). Similar patterns of baseline characteristic differences were also seen in the two-dose comparison cohorts (Fig. 2 and Supplementary Table 3). All covariates were balanced after inverse probability weighting (IPW) with an absolute standardised mean difference < 0.1, including the date (calendar week) of vaccination.

### Incidence and hazard ratio

Over the 14,630 and 20,714 person-years of follow-up for the one dose BNT162b2 and ChAdOx1 recipient, 200 and 261 people tested positive for SARS-CoV-2, equivalent to incidence rates (IR) of 13.7 and 12.6/1,000 person-years, respectively, and an unadjusted hazard ratio (HR) of 1.08 (95% 0.90 - 1.30). After IPW, the HR changed to 0.72 (95% 0.58 - 0.88), favouring BNT162b2 in the overall population (Table 1). In contrast, the incidences of Covid-19 hospitalisation were similar among the one dose BNT162b2 (IR: 3.07 per 1,000 person-years) and ChAdOx1 (IR: 2.17 per 1,000 person-years) cohorts, with no noticeable differences between both vaccine groups: adjusted/weighted HR 0.87 (95% 0.53 - 1.41).

**Table 1:** Incidence rate and hazard ratio of Covid-19 infection and hospitalisation following one and two doses of vaccines.

	BNT162b2			ChAdOx1				
Covid-19 outcomes	Total PYs at risk	Cases	Incidence, per 1000 PYs	Total PYs at risk	Cases	Incidence, per 1000 PYs	Unadjusted HR	Adjusted HR
One dose								
Infection	14630	200	13.70	20,714	261	12.60	1.08 (0.90– 1.30)	0.72 (0.58–0.88)
Hospitalisation	14654	45	3.07	20,746	45	2.17	1.41 (0.95–2.09)	0.87 (0.53–1.41)
Two doses								
Infection	34991	1361	38.90	44,084	2497	56.60	0.72 (0.57–0.91)	0.70 (0.65–0.75)
Hospitalisation	35176	122	3.47	44,421	202	4.55	0.66 (0.62–0.71)	0.71 (0.55–0.90)

*PYs* person-years, *HR* hazard ratio.

After the second dose, 1361/34,991 person-years (IR: 38.9 per 1000 person-years) and 2,497/44,084 person-years (IR: 56.6 per 1000 person-years) were identified positive for SARS-CoV-2 among BNT162b2 and ChAdOx1 recipients respectively. Unadjusted (0.72, 95% 0.57–0.91) and adjusted HR (0.70, 95% 0.65–0.75) were almost identical, favouring BNT162b2 (Table 1). The rates of Covid-19 hospitalisation remained low in both cohorts, but higher amongst ChAdOx1 (IR: 4.55 per 1000 person-years) compared to BNT162b2 recipients (IR: 3.47 per 1000 person-years), with adjusted HR of 0.71 (95% 0.55–0.90) favouring BNT162b2.

### Kaplan–Meier curve of Covid-19 infection and hospitalisation

**Fig. 3:**
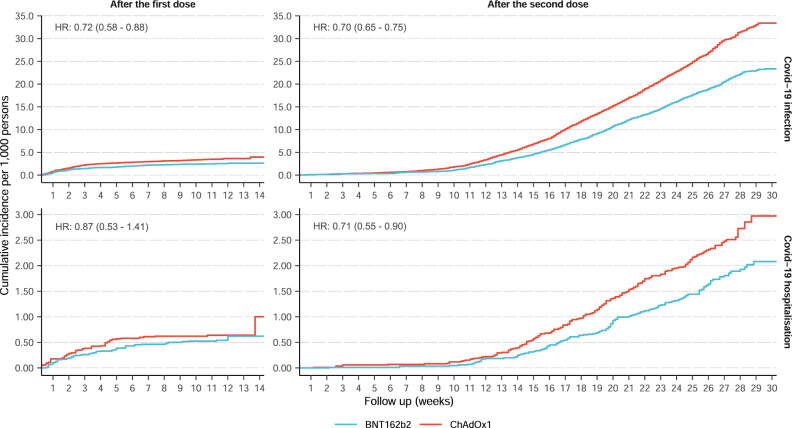
Kaplan-Meier cumulative incidence curves of Covid-19 infection and hospitalisation after one and two doses of vaccines. The different Y-axis scales for Covid-19 infection and hospitalisation outcomes. The follow-up was up to 14 weeks after the first dose and 30 weeks after the second dose of the vaccine. HR hazard ratio.

Kaplan–Meier curves stratified by vaccine depicted similar trends between Covid-19 infection and hospitalisation. The cumulative incidence after the first dose increased rapidly in the early follow-up but flattened later until 14 weeks after vaccination (Fig. 3), corresponding to the calendar period from January to March 2021 (Supplementary Fig. 1). Conversely, the trend of cumulative incidence was reversed for the two-dose cohorts, with a substantial increase starting 12 weeks after the second dose (Fig. 3), corresponding to the calendar period from June to October 2021 (Supplementary Fig. 1). Changes in community transmission over time in the general population of England and among UK Biobank participants are shown in Supplementary Figure 1.

### Comparative effectiveness across sub-populations and over-time

The risk of Covid-19 infection was consistently lower in people receiving two doses of BNT162b2 compared to those receiving two doses of ChAdOx1 across all stratas. HRs ranged from 0.65 (95% 0.59–0.71) for females to 0.80 (95% 0.70–0.90) for oldest-old adults.

**Fig. 4:**
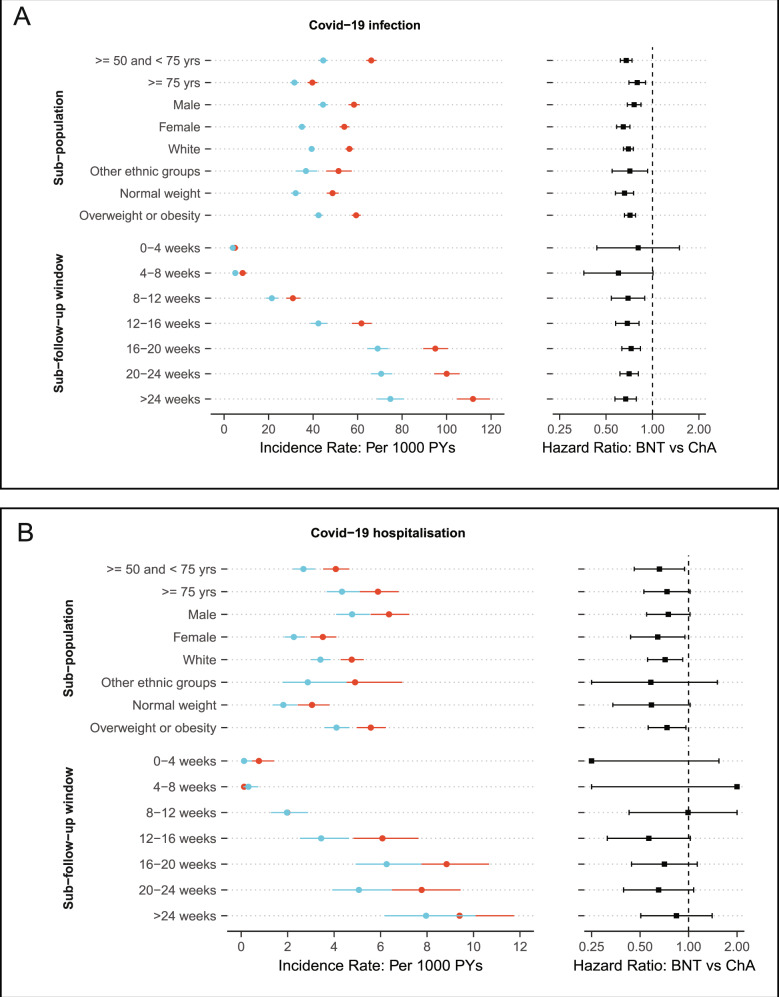
Comparative effectiveness of two doses BNT162b2 vs ChAdOx1 vaccine on Covid-19 infection and hospitalisation across key subgroups and over time. The different X-axis range of incidence rate for the Covid-19 infection (**A**) and hospitalisation outcome (**B**). Hazard ratios for Covid-19 hospitalisation were truncated at 0.2 (lowest) and 2 (highest). Blue and red points indicated BNT162b2 and ChAdOx1 respectively. PYs: person-years. BNT: BNT162b2. ChA: ChAdOx1.

Notably, incidence rates of Covid-19 infection increased substantially during the study period, from 3.93 and 4.87 per 1000 person-years at the 0–4-week window to 74.74 and 111.87 per 1,000 person-years at the >24-week window in BNT162b2 and ChAdOx1 cohorts, respectively. However, as reflected by HRs, the comparative risks were stable for at least 6 months (Fig. 4A). Although a similar pattern was observed for Covid-19 hospitalisation, power was limited for the time-split analysis, in particular for the 0–4-week window (HR: 0.16, 95% 0.02–1.54) and 4–8-week window (HR: 2.48, 95% 0.16–39.66), due to the extremely rare events observed in the first few weeks after the second vaccination (Fig. 4B).

### Negative control and sensitivity analyses

Adjusted hazard ratios for proposed negative clinical outcomes according to vaccine received among the one dose cohort were 1.01 (0.95–1.17) for limb pain, 0.93 (0.82–1.05) for fracture, and 0.88 (0.51–1.51) for peptic ulcer.

The main results were consistent in the propensity score-matched cohorts with slightly wider confidence intervals (Supplementary Table 4).

## Discussion

Among adults aged 50 years and older, we found that people receiving two doses of BNT162b2 vaccine had 30% lower risks of both Covid-19 diagnosis and related hospital admission, compared with those vaccinated with ChAdOx1. A similar difference was also observed among the one dose recipients, yet, only for Covid-19 diagnosis. Notably, the two-dose comparative effectiveness was consistent across several high-risk groups, such as oldest-old^7^, male^8^, ethnic minority^9^ and those with overweight or obesity^10^, and persisted over time for over six months, verifying the hypothesis from a few recent preprint studies^11,12^.

To date, only two Phase 2 randomized controlled trial has directly compared the efficacy of mRNA with adenovirus-based Covid-19 vaccines by using immunogenic endpoints. Liu et al.^13^ found that the BNT162B2-BNT162B2 appeared more immunogenic than the ChAdOx1-ChAdOx1 schedule, with higher levels of antibodies at 28 days after the first and second dose. A similar finding was reported in the Borobia et al. study comparing the heterologous BNT162B2-ChAdOx1 and the homogenous ChAdOx1-ChAdOx1 vaccine regimens^14,15^. In line with this evidence, our study showed a lower risk of Covid-19 outcomes in people vaccinated with BNT162b2 overall and across subgroups.

However, a recent preprint study by Hulme et al.^16^ showed no difference among recipients of these two vaccines regarding the risk of SARS-CoV-2 positive test, Covid-19 related accident & emergency attendance and hospital admission. Several fundamental factors might explain the disagreement with our results. First, Hulme’s study only included health and social care workers who are more likely to be exposed to SARS-CoV-2 than our cohort: a community-based “healthy volunteer” population^17^, which was corroborated by the higher infection rate in their study (58 per 1000 person-years) compared to ours (~13 per 1000 person-years) during the first few weeks following the first dose. Second, both ours and Hulme’s studies found that, on average, rollout of BNT162B2 happened earlier than ChAdOx1. Given the variation in community transmission over time in England, insufficient control for vaccination date could result in biased estimates of their comparative effect. Informed by this, we observed a substantial change of hazard ratio after the complete alignment of the first vaccination date using the weighting approach when the background transmission varied substantially (Supplementary Figure 1, Exposure anchor period). In contrast, adjusting/weighting the second vaccination date had little impact on the estimate, likely due to the relatively low and stable virus circulation at that period (Supplementary Figure 1, Exposure anchor period). Third, Hulme’s risk assessment period started from the first dose (January to February 2021) and ended on 13 June 2021, reflecting a mixed effect of one and two doses of vaccines.

The leading challenges in estimating vaccine effectiveness with observational data lies in confounding by indication and potentially differential testing rates between exposed vaccinated and unvaccinated populations^18,19^. However, our study minimized the impact of such differences by comparing two vaccines and restricting the analysis to a period when both vaccines were available and had a similar national delivery. UK data are ideal for comparative effectiveness research into Covid-19 vaccines, as both BNT162b2 and ChAdOx1 were rolled out simultaneously for the target population (adults ≥ 50 years) included in our analyses^20–22^.

However, a few limitations remain. Information on some participants’ characteristics such as socioeconomic status was collected ten years ago and may have changed since then. However, given that all people at the cohort recruitment were already middle-aged or older adults (40–69 years old), we expected any changes in those features are likely to be minor or unrelated to the choice of vaccine types. Admittedly, misclassification of covariates could bias the genuine association towards the null and lead to underestimating the comparative vaccine effectiveness. Also, our data are limited to participants aged 50 or above. However, this is precisely the most vulnerable population in the Covid-19 pandemic.

Our study has several unique strengths. First, the granularity of UK Biobank data and comprehensive linkage to external data sources allowed us to measure and control for an extensive array of confounders, including demographics, socio-economic deprivation, comorbidity, and medication usage. Our negative control outcome analyses provided reassurance that no significant residual confounding remained after adjusting the mentioned covariates using IPW methods. Secondly, the proposed study outcomes (Covid-19 and related hospitalization) were identified through linkage to official national databases of tests and hospital inpatient data, minimising the risk of misclassification. Finally, the sample size of our cohort triplicated that of the largest phase III trials, enabling us to detect differences in hospital admission rates, which seemed underpowered in randomized trials.

Our findings support evidence from pivotal trials suggesting that BNT162b2 provides additional protection against Covid-19 and hospitalisation than ChAdOx1 vaccination. For the first time, we demonstrated that this comparative effectiveness endured over six months when the Delta variant was predominant, and community transmission kept increasing in the UK. These findings highlight the importance of continuous monitoring of the effectiveness of different vaccines against emerging SARS-CoV-2 variants to inform future booster campaigns and vaccine combinations strategies.

## Methods

### Study population and design

We used data from the UK Biobank (UKBB) cohort, a prospective study of approximately 500,000 individuals aged between 40 and 69 at baseline recruited in 2007-2010 from England (89%), Scotland (7%) and Wales (4%). All participants provided comprehensive information on socio-demographic, lifestyle, and health-related factors, which has been described in detail elsewhere^23^. The initial protocol of the UKBB study is available (https://www.ukbiobank.ac.uk/media/gnkeyh2q/study-rationale.pdf), which has ethical approval from its own Ethics Advisory Committee (EAC) (https://www.ukbiobank.ac.uk/ethics/). The participants gave informed consent and public involvement are detailed online (https://www.ukbiobank.ac.uk/learn-more-about-uk-biobank). This project was granted under the application of 65397.

In this study, we analysed participants from England, as vaccination data for Scotland and Wales were not available. Although vaccination started in early December 2020 in the UK, we restricted our analyses to periods when both vaccines were rolled out to maximise comparability in individual- and population-level characteristics, including indication for vaccination, ongoing public health restrictions and predominant virus variants. With this in mind, we enrolled one-dose and two-dose Covid-19 vaccine cohorts covering from Jan 11, 2021, to Feb 28, 2021, and from March 22, 2021, to May 9, 2021, respectively. Participants with primary care records generated using the TTP software, which did not contain a specific vaccine type, were excluded.

### Data sources

Several external data sources have been linked to UK Biobank to enable Covid-19 research, including primary care electronic health records, hospital admissions data from Hospital Episode Statistics (HES), and Covid-19 tests from Public Health England (PHE)^24^. Data coverage for each data source is listed in the Supplementary Methods.

### Vaccination status

Vaccination status was obtained from GP prescription records, including the date of receipt of each dose and the vaccine brand. We used dm+d codes (Dictionary of medicines and devices used across the UK’s National Health Service) to identify BNT162b2 [39115611000001103] and ChAdOx1 [39114911000001105] Covid-19 vaccination.

### Study outcomes

In the UK, people at any age with any of these three Covid-19 symptoms (a high temperature, a new and continuous cough, or loss or change in the sense of smell or taste) are recommended to take a free Polymerase-chain-reaction (PCR) test by ordering a self-swab home PCR test kit or booking an appointment at a walk-in or drive-through test site. People could also access this service if they were at high risk of infection, e.g., close contact with a case (see https://www.gov.uk/get-coronavirus-test for details). All these tests were recorded by PHE and linked to UK Biobank, providing information on test dates and results. In this study, we defined infection as having a positive PCR test for SARS-CoV-2; and Covid-19 hospitalization (infection requiring hospital admission) based on the UKBB derived algorithm described in the Supplementary Methods.

### Covariates

We assessed multiple characteristics potentially associated with Covid-19 risk and/or vaccination, therefore considered as confounders. Study covariates included socio-demographics (age, sex, ethnicity), socio-economic status (index of multiple deprivations, education levels), physical measurements (body mass index), healthcare resource use (prescribed medications and the number of hospital admissions three years before the vaccination date), and comorbidities. The calendar week of receipt of the Covid-19 vaccine was also included as it affected the probability of receiving different vaccines and infection risk (through changes in community transmission level).

### Statistical analyses

The outcome risk assessment window for the one-dose cohort went from receiving the first dose to the earliest of outcome occurrence, receiving the second dose, or 14 weeks after the vaccination. For the two-dose cohort, follow-up was from receiving the second dose to outcome occurrence or end of the study (18/10/2021).

We used the propensity score-based inverse probability of treatment weighting (IPW) to minimise confounding^25^. The specification of propensity score modelling was described in Supplementary Methods. We generated Kaplan-Meier plots to depict the cumulative incidence of outcome over time in each cohort. We applied Cox proportional hazards regression with robust variance estimators to derive average hazard ratio (HR) and calculated incidence rates using weighted counts and follow-up time. We assessed the proportionality of hazards in the Cox models by visually inspecting scaled Schoenfeld residuals.

To evaluate for potential heterogeneity of the comparative effectiveness among specific demographic subgroups and overtime after the second dose, we performed several secondary analyses by including multiplicative interaction terms between the vaccine types and the following categories separately: age (50–75 years or > 75 years), sex (male or female), ethnicity (white or other ethnic groups), BMI ( < 25 vs. ≥ 25), and four weeks’ consecutive time intervals.

We conducted the negative control experiment to assess potential residual (unobserved) confounding. Three clinical outcomes (limb pain, fracture, and peptic ulcer events) were pre-specified and should not be associated with vaccination status (BNT162b2 vs ChAdOx1) if potential confounders have been adequately controlled for.

Finally, we performed a sensitivity analysis using propensity score 1:1 matching without replacement. Specifically, we set a caliper of width equal to 0.2 of the standard deviation of the logit of the propensity score^26–28^. We reported the standard 95% confidence intervals and used R version 4.0.4 for all analyses.

### Reporting summary

Further information on research design is available in the Nature Research Reporting Summary linked to this article.

## Supplementary information

Supplementary Information

Reporting Summary

## Data Availability

Auxiliary and summary data generated from the analyses are available in the supplementary file. Source data of UK Biobank are assessable by registering and applying at http://ukbiobank.ac.uk/register-apply. The data for weekly Covid-19 cases and SARS-CoV-2 Variants of Concern in England were from the Public Health England (https://coronavirus.data.gov.uk/).

## Code availability

The code used for this study has been deposited in the git repository (https://github.com/xjq8065524/NC_comparative_vaccines_effectiveness).
