# Supplementary material for: Comparative effectiveness of the BNT162b2 and ChAdOx1 vaccines against Covid-19 in people over 50
Source: Nat Commun. 2022 Mar 21;13:1519. doi: 10.1038/s41467-022-29159-x (PMC8938429; doi:10.1038/s41467-022-29159-x)
Supplement: Supplementary file 1 — Supplementary Information [file 41467_2022_29159_MOESM1_ESM.pdf]

Supplementary items

Contents

Supplementary Figure 1: The transmission dynamics of SARS-CoV-2 (A) and its dominant variants (B) during the study period. ... 2

Supplementary Table 2: Baseline characteristics of the one-dose cohorts. ... 3

Supplementary Table 3: Baseline characteristics of the two-doses cohorts. ... 6

Supplementary Table 4: Sensitivity analysis (propensity score matching) ... 9

Supplementary Methods: Sources and timelines for data availability ... 10

Supplementary Methods: Construction of the inpatient indicator for UKBB participants ... 11

Supplementary Methods: Propensity score modelling and inverse probability of treatment weighting ... 12

Supplementary Figure 1: The transmission dynamics of SARS-CoV-2 (A) and its dominant variants (B) during the study period.

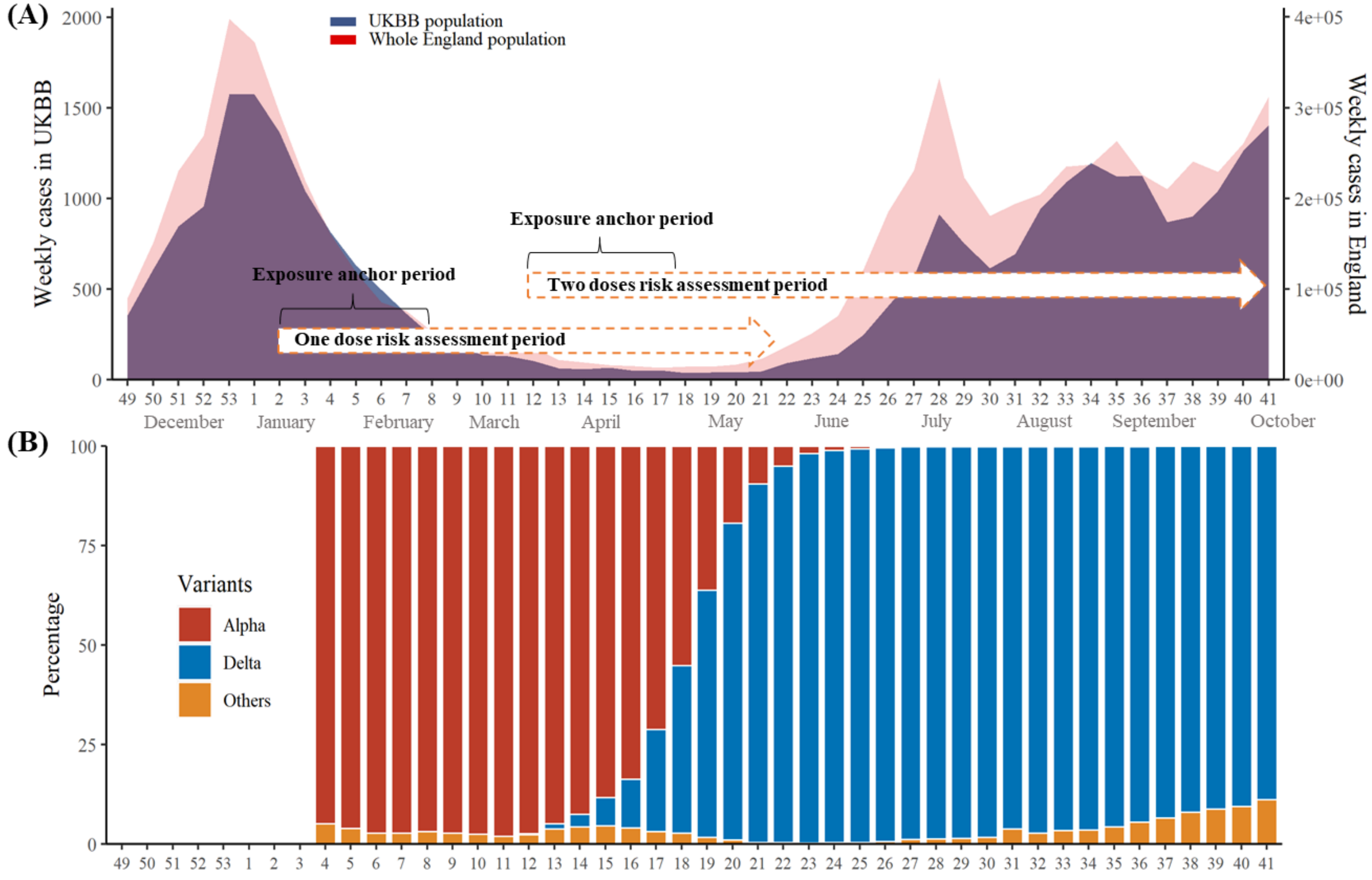

Notes: The data for weekly Covid-19 cases and SARS-CoV-2 variants of concern in England were from the Public Health England (https://coronavirus.data.gov.uk/).

Supplementary Table 2: Baseline characteristics of the one-dose cohorts.

	Before weighting		After weighting	
	BNT162b2	ChAdOx1	BNT162b2	ChAdOx1
Number	70097	98551	167938	167917
**Previous infection (%)**	1843 (2.6)	2649 (2.7)	4635.7 (2.8)	4662.1 (2.8)
**Vaccination calendar week (%)**				
Week2	12224 (17.4)	3127 (3.2)	15716.6 (9.4)	14681.5 (8.7)
Week3	15591 (22.2)	15728 (16.0)	31194.2 (18.6)	31220.5 (18.6)
Week4	14783 (21.1)	16990 (17.2)	32056.9 (19.1)	32096.3 (19.1)
Week5	7655 (10.9)	24264 (24.6)	31629.9 (18.8)	31957.0 (19.0)
Week6	7573 (10.8)	19492 (19.8)	26858.2 (16.0)	27070.1 (16.1)
Week7	5907 (8.4)	11282 (11.4)	16731.3 (10.0)	17059.5 (10.2)
Week8	6364 (9.1)	7668 (7.8)	13751.8 (8.2)	13832.5 (8.2)
**Demographics**				
Age, mean (sd)	71.35 (7.21)	71.06 (6.02)	71.09 (6.68)	71.11 (6.48)
Sex, male (%)	31203 (44.5)	43432 (44.1)	74111.1 (44.1)	74156.6 (44.2)
BMI, mean (sd)	27.56 (4.78)	27.46 (4.76)	27.51 (4.77)	27.51 (4.78)
**Ethnicity (%)**				
Asian or Asian British	1479 (2.1)	1563 (1.6)	3112.1 (1.9)	3085.9 (1.8)
Black or Black British	1136 (1.6)	1138 (1.2)	2311.8 (1.4)	2220.0 (1.3)
Chinese	224 (0.3)	263 (0.3)	478.5 (0.3)	481.6 (0.3)
Do not know	30 (0.0)	41 (0.0)	71.5 (0.0)	75.7 (0.0)
Mixed	397 (0.6)	460 (0.5)	851.1 (0.5)	840.7 (0.5)
Other ethnic group	657 (0.9)	703 (0.7)	1374.9 (0.8)	1363.6 (0.8)
Prefer not to answer	2248 (3.2)	3102 (3.1)	5267.6 (3.1)	5257.8 (3.1)
White	63926 (91.2)	91281 (92.6)	154471.3 (92.0)	154592.2 (92.1)
**Education (%)**				
None of the above	12247 (17.5)	16502 (16.7)	28750.7 (17.1)	28763.7 (17.1)
Prefer not to answer	2101 (3.0)	2323 (2.4)	4455.3 (2.7)	4320.0 (2.6)
College or university degree	21484 (30.6)	31157 (31.6)	52075.8 (31.0)	52190.1 (31.1)
A levels as levels or equivalent	7498 (10.7)	11054 (11.2)	18481.5 (11.0)	18474.9 (11.0)
O levels gcs es or equivalent	14812 (21.1)	20890 (21.2)	35573.0 (21.2)	35668.3 (21.2)

Cs es or equivalent	3473 (5.0)	4776 (4.8)	8245.5 (4.9)	8268.9 (4.9)
Nvq or hnd or hnc or equivalent	4605 (6.6)	6501 (6.6)	11099.1 (6.6)	11120.0 (6.6)
Other professional qualifications	3877 (5.5)	5348 (5.4)	9257.9 (5.5)	9111.5 (5.4)
**Indices of Multiple Deprivation, mean (sd)**				
Townsend deprivation index	17.70 (13.96)	16.49 (13.37)	17.08 (13.57)	17.01 (13.73)
Income score	0.12 (0.10)	0.11 (0.09)	0.11 (0.09)	0.11 (0.10)
Employment score	0.09 (0.06)	0.08 (0.06)	0.09 (0.06)	0.09 (0.06)
Health score	-0.11 (0.89)	-0.18 (0.88)	-0.13 (0.89)	-0.14 (0.89)
Education score	14.61 (14.90)	14.13 (14.83)	14.40 (14.74)	14.39 (15.02)
Housing score	19.94 (9.95)	19.80 (9.98)	19.70 (9.84)	19.69 (9.97)
Crime score	-0.04 (0.78)	-0.09 (0.77)	-0.07 (0.77)	-0.07 (0.77)
**Medications (past three years)**				
Lipid lowering drugs	33093 (47.2)	43266 (43.9)	76151.4 (45.3)	75957.6 (45.2)
RAS inhibitors	21956 (31.3)	29023 (29.4)	50602.1 (30.1)	50609.0 (30.1)
Other anti-hypertensives	11158 (15.9)	14517 (14.7)	25459.6 (15.2)	25458.1 (15.2)
Proton pump inhibitors	30579 (43.6)	41572 (42.2)	72068.6 (42.9)	71994.1 (42.9)
Diabetes medicines	6666 (9.5)	8288 (8.4)	14982.9 (8.9)	14899.5 (8.9)
Antidepressants	14515 (20.7)	20199 (20.5)	34836.6 (20.7)	34987.6 (20.8)
Systemic glucocorticoids	9282 (13.2)	12919 (13.1)	22129.9 (13.2)	22245.6 (13.2)
Antithrombotic	11799 (16.8)	15061 (15.3)	26856.6 (16.0)	26832.6 (16.0)
Anticoagulants	3110 (4.4)	4020 (4.1)	7099.6 (4.2)	7130.8 (4.2)
Immunosuppressants excl corticosteroids	1447 (2.1)	2017 (2.0)	3448.5 (2.1)	3492.0 (2.1)
Antineoplastic agents	180 (0.3)	251 (0.3)	413.7 (0.2)	413.4 (0.2)
**Hospital admission frequency (past three years)**	1.38 (5.52)	1.34 (5.25)	1.37 (6.36)	1.37 (5.12)
**Charlson comorbidity components (past years)**				
Diabetes (uncomplicated)	9193 (13.1)	11657 (11.8)	20797.9 (12.4)	20800.9 (12.4)
COPD	14156 (20.2)	19433 (19.7)	33547.1 (20.0)	33504.9 (20.0)
Chronic kidney disease	7449 (10.6)	9368 (9.5)	16714.7 (10.0)	16713.1 (10.0)
Congestive heart failure	1473 (2.1)	1893 (1.9)	3344.1 (2.0)	3370.8 (2.0)
Cancer	10807 (15.4)	14483 (14.7)	25028.3 (14.9)	24975.7 (14.9)
Dementia	681 (1.0)	959 (1.0)	1731.3 (1.0)	1827.9 (1.1)
Cerebrovascular disease	2799 (4.0)	3498 (3.5)	6356.5 (3.8)	6324.4 (3.8)
Diabetes (end-organ damage)	3821 (5.5)	4755 (4.8)	8556.7 (5.1)	8526.5 (5.1)

Peripheral vascular disease	1459 (2.1)	1857 (1.9)	3276.3 (2.0)	3287.2 (2.0)
Liver disease (moderate to severe)	279 (0.4)	384 (0.4)	631.3 (0.4)	646.1 (0.4)
Peptic ulcer	2151 (3.1)	2877 (2.9)	5081.0 (3.0)	5018.4 (3.0)
Rheumatoid arthritis	3118 (4.4)	4177 (4.2)	7266.1 (4.3)	7265.0 (4.3)
Myocardial infarction	2573 (3.7)	3254 (3.3)	5890.7 (3.5)	5866.9 (3.5)
AIDS	137 (0.2)	183 (0.2)	320.8 (0.2)	310.5 (0.2)
Malignant cancer	370 (0.5)	502 (0.5)	859.2 (0.5)	836.4 (0.5)
Liver disease (mild)	452 (0.6)	650 (0.7)	1105.4 (0.7)	1110.2 (0.7)
Hemiplegia	98 (0.1)	147 (0.1)	263.2 (0.2)	250.8 (0.1)

Supplementary Table 3: Baseline characteristics of the two-doses cohorts.

	Before weighting		After weighting	
	BNT162b2	ChAdOx1	BNT162b2	ChAdOx1
Number	67813	89030	156301	156952
**Previous infection (%)**	2539 (2.9)	1818 (2.7)	4518.5 (2.9)	4498.7 (2.9)
**Vaccination calendar week (%)**				
Week12	8299 (12.2)	3972 (4.5)	12366.1 (7.9)	12606.7 (8.0)
Week13	13558 (20.0)	4373 (4.9)	18073.8 (11.6)	17812.4 (11.3)
Week14	12976 (19.1)	13900 (15.6)	26803.0 (17.1)	26791.8 (17.1)
Week15	13365 (19.7)	16445 (18.5)	29957.9 (19.2)	29935.1 (19.1)
Week16	6892 (10.2)	21591 (24.3)	28278.7 (18.1)	28468.5 (18.1)
Week17	7037 (10.4)	16945 (19.0)	23698.1 (15.2)	23941.3 (15.3)
Week18	5686 (8.4)	11804 (13.3)	17123.5 (11.0)	17396.8 (11.1)
**Demographics**				
Age, mean (sd)	72.47 (6.97)	71.51 (5.98)	71.80 (6.74)	71.81 (6.18)
Sex, male (%)	29825 (44.0)	39250 (44.1)	68880.5 (44.1)	69199.6 (44.1)
BMI, mean (sd)	27.47 (4.67)	27.46 (4.74)	27.50 (4.72)	27.50 (4.75)
**Ethnicity (%)**				
Asian or Asian British	1279 (1.9)	1348 (1.5)	2656.8 (1.7)	2641.7 (1.7)
Black or Black British	924 (1.4)	969 (1.1)	1957.9 (1.3)	1921.4 (1.2)
Chinese	185 (0.3)	225 (0.3)	405.8 (0.3)	409.4 (0.3)
Do not know	26 (0.0)	37 (0.0)	68.3 (0.0)	66.4 (0.0)
Mixed	345 (0.5)	408 (0.5)	730.0 (0.5)	735.3 (0.5)
Other ethnic group	560 (0.8)	613 (0.7)	1182.2 (0.8)	1171.0 (0.7)
Prefer not to answer	2143 (3.2)	2737 (3.1)	4807.1 (3.1)	4881.0 (3.1)
White	62351 (91.9)	82693 (92.9)	144492.9 (92.4)	145126.1 (92.5)
**Education (%)**				
None of the above	12648 (18.7)	15435 (17.3)	28036.2 (17.9)	28234.6 (18.0)
Prefer not to answer	2031 (3.0)	2116 (2.4)	4097.4 (2.6)	4059.5 (2.6)
College or university degree	20513 (30.2)	27567 (31.0)	47638.2 (30.5)	47836.8 (30.5)
A levels as levels or equivalent	7069 (10.4)	9848 (11.1)	16888.3 (10.8)	16951.9 (10.8)
O levels gcs es or equivalent	14183 (20.9)	19030 (21.4)	33181.6 (21.2)	33314.8 (21.2)

Cs es or equivalent	2970 (4.4)	4142 (4.7)	7166.0 (4.6)	7180.5 (4.6)
Nvq or hnd or hnc or equivalent	4440 (6.5)	5916 (6.6)	10415.2 (6.7)	10483.2 (6.7)
Other professional qualifications	3959 (5.8)	4976 (5.6)	8878.2 (5.7)	8891.1 (5.7)
Indices of Multiple Deprivation, mean (sd)				
Townsend deprivation index	17.35 (13.69)	16.39 (13.27)	17.00 (13.51)	16.97 (13.62)
Income score	0.11 (0.09)	0.11 (0.09)	0.11 (0.09)	0.11 (0.09)
Employment score	0.09 (0.06)	0.08 (0.06)	0.09 (0.06)	0.09 (0.06)
Health score	-0.12 (0.89)	-0.18 (0.88)	-0.14 (0.88)	-0.14 (0.88)
Education score	14.39 (14.77)	14.10 (14.80)	14.35 (14.72)	14.35 (14.96)
Housing score	19.85 (9.90)	19.66 (9.92)	19.64 (9.80)	19.66 (9.97)
Crime score	-0.05 (0.78)	-0.09 (0.77)	-0.07 (0.77)	-0.07 (0.77)
**Medications (past three years)**				
Lipid lowering drugs	33138 (48.9)	40248 (45.2)	73220.9 (46.8)	73457.8 (46.8)
RAS inhibitors	21768 (32.1)	26794 (30.1)	48369.8 (30.9)	48521.5 (30.9)
Other anti-hypertensives	11297 (16.7)	13489 (15.2)	24636.9 (15.8)	24732.6 (15.8)
Proton pump inhibitors	30074 (44.3)	38128 (42.8)	68201.9 (43.6)	68396.9 (43.6)
Diabetes medicines	6168 (9.1)	7556 (8.5)	13866.2 (8.9)	13854.3 (8.8)
Antidepressants	13877 (20.5)	18277 (20.5)	32391.9 (20.7)	32511.1 (20.7)
Systemic glucocorticoids	9126 (13.5)	11585 (13.0)	20797.3 (13.3)	20784.2 (13.2)
Antithrombotic	11903 (17.6)	14077 (15.8)	26024.1 (16.6)	26049.1 (16.6)
Anticoagulants	3392 (5.0)	3969 (4.5)	7289.0 (4.7)	7271.8 (4.6)
Immunosuppressants excl corticosteroids	1357 (2.0)	1823 (2.0)	3216.2 (2.1)	3222.4 (2.1)
Antineoplastic agents	185 (0.3)	232 (0.3)	411.4 (0.3)	398.8 (0.3)
**Hospital admission frequency** (**past three years**)	1.39 (5.82)	1.32 (5.02)	1.37 (6.36)	1.36 (4.89)
**Charlson comorbidity components (past years)**				
Diabetes (uncomplicated)	8663 (12.8)	10667 (12.0)	19443.3 (12.4)	19495.5 (12.4)
COPD	13737 (20.3)	17715 (19.9)	31566.4 (20.2)	31582.3 (20.1)
Chronic kidney disease	7746 (11.4)	8924 (10.0)	16513.0 (10.6)	16558.8 (10.6)
Congestive heart failure	1548 (2.3)	1814 (2.0)	3355.0 (2.1)	3359.2 (2.1)
Cancer	10937 (16.1)	13601 (15.3)	24305.2 (15.6)	24366.2 (15.5)
Dementia	820 (1.2)	1004 (1.1)	1871.1 (1.2)	1942.2 (1.2)
Cerebrovascular disease	2860 (4.2)	3327 (3.7)	6199.3 (4.0)	6173.1 (3.9)
Diabetes (end-organ damage)	3638 (5.4)	4411 (5.0)	8099.5 (5.2)	8084.2 (5.2)

Peripheral vascular disease	1513 (2.2)	1760 (2.0)	3258.4 (2.1)	3256.7 (2.1)
Liver disease (moderate to severe)	258 (0.4)	348 (0.4)	605.6 (0.4)	606.2 (0.4)
Peptic ulcer	2190 (3.2)	2684 (3.0)	4912.0 (3.1)	4898.0 (3.1)
Rheumatoid arthritis	3225 (4.8)	3939 (4.4)	7172.6 (4.6)	7153.7 (4.6)
Myocardial infarction	2541 (3.7)	3053 (3.4)	5663.6 (3.6)	5663.5 (3.6)
AIDS	131 (0.2)	164 (0.2)	294.3 (0.2)	288.0 (0.2)
Malignant cancer	352 (0.5)	462 (0.5)	789.2 (0.5)	804.3 (0.5)
Liver disease (mild)	421 (0.6)	579 (0.7)	1005.7 (0.6)	1022.9 (0.7)
Hemiplegia	90 (0.1)	123 (0.1)	225.5 (0.1)	222.0 (0.1)

Supplementary Table 4: Sensitivity analysis (propensity score matching)

	BNT162b2 vs ChAdOx1			
	Number	Events	Incidence	HRs
After first-dose				
Covid-19 infection	58676/ 58676	154/ 193	12.6/ 15.7	0.80 (0.65 - 0.99)
Covid-19 hospitalization	58676/ 58676	29/ 32	2.36/ 2.60	0.91 (0.55 - 1.51)
After second-dose				
Covid-19 infection	54422/ 54422	1089/ 1582	39.5/ 57.6	0.69 (0.63 - 0.74)
Covid-19 hospitalization	54422/ 54422	93/ 142	3.35/ 5.13	0.65 (0.50 - 0.85)

Supplementary Methods: **Sources and timelines for data availability**

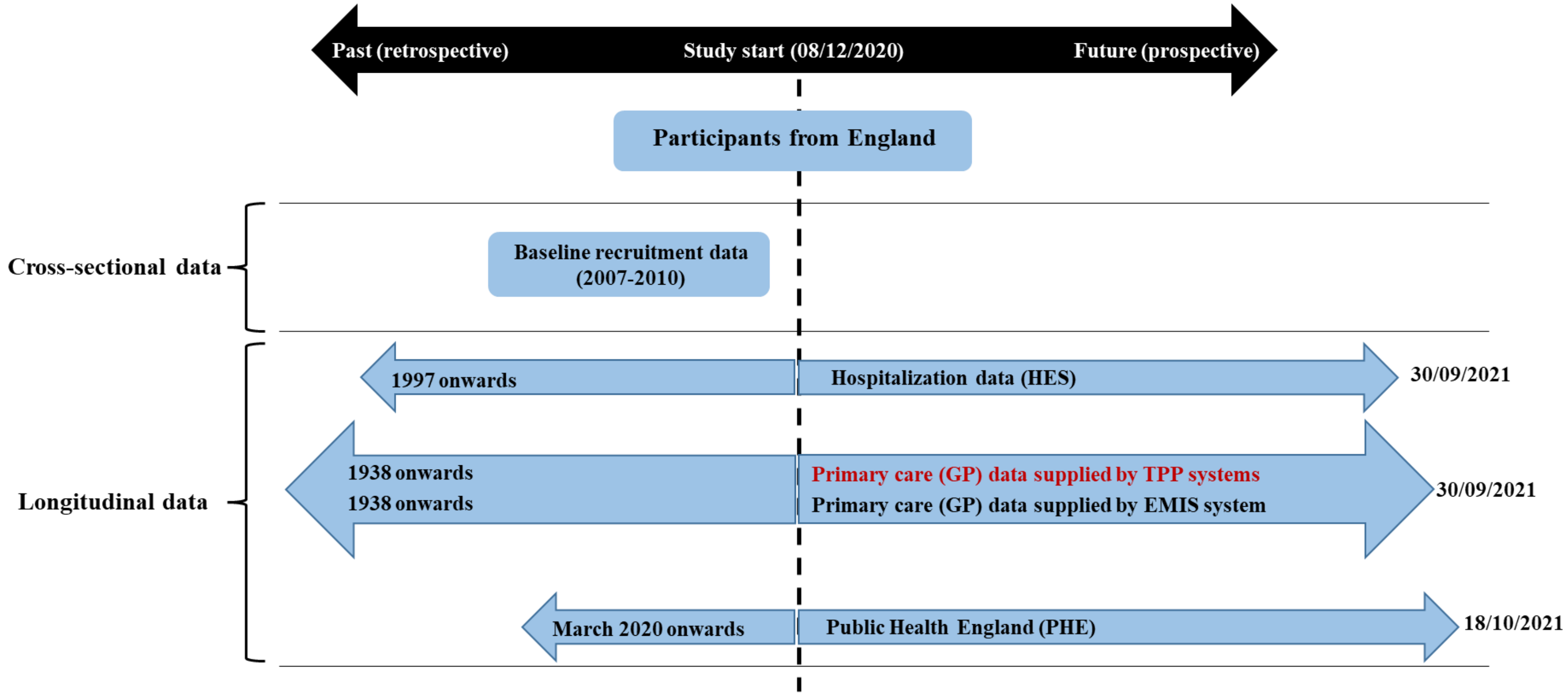

The baseline recruitment data includes detailed demographic, socioeconomic, lifestyle factors, and physical activities information. The HES data includes hospital administration, operations and diagnoses information. The GP data includes diagnoses and prescription information. Of note, the Covid-19 vaccination has been integrated into EMIS GP's prescription records but not into the TPP. The PHE data includes Covid-19 test results.

Supplementary Methods: Construction of the inpatient indicator for UKBB participants

The construction of the "origin" field is based on information provided on the specimen request form. If the specimen was marked as being from an acute (emergency) care provider, an A&E department, an inpatient location, or resulted from health care associated infection, it is recorded by PHE as an inpatient sample. Tests marked as being from "Healthcare Worker Testing" are never recorded as inpatient samples, though some may also carry an acute flag.

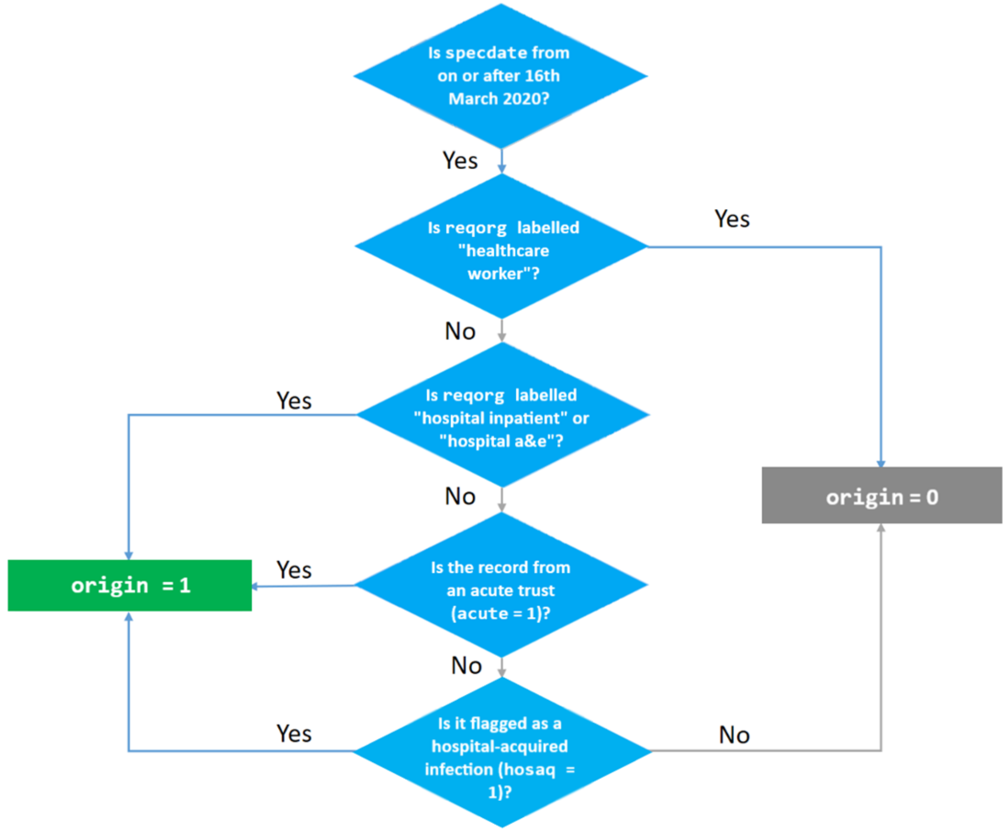

The aim of designating inpatient status for the SARS-CoV-2 test was to indicate severity of Covid-19 infection. SARS-CoV-2 tests taken in hospital can be undertaken for several reasons including symptomatic patients requiring hospital admission, or general inpatient screening, which includes asymptomatic patients. Furthermore, the algorithm used to flag inpatient status may not necessarily indicate inpatient care in all cases. For example, some tests flagged as coming from "acute" trusts will likely not be inpatients, since these trusts may also perform tests on behalf of GPs and others, and tests requested by A&E may be for patients who are not then admitted.

The flow chart below illustrates the logic used by PHE to generate an indicator of whether a test result was obtained from a hospital inpatient or not (depicted as the "origin" field in the covid19_result table). The fields used to construct the "origin" field have also been released to enable researchers to replicate it and construct their own alternatives if desired.

PHE's designation of inpatient status can be compared to hospital episodes statistics (HES) dates of admission and discharge made by NHS trusts. A comparison between SARS-CoV-2 positive inpatient status versus inpatient diagnosis codes (ICD-10 diagnosis codes U071 or U072) for Covid-19 (from HES) can also be made. For further details, see the following webpage: https://news.bugbank.uk/2020/08/identifying-inpatients-comparison-to.html.

Supplementary Methods: **Propensity score modelling and inverse probability of treatment weighting**

We calculated propensity scores for a vaccination with BNT162b2 against ChAdOx1 using logistic regression. The variables included in the model were previous Covid-19 infection status (binary), age on the vaccination date (continuous linear), sex (binary), ethnicity (categorial, collected at the UK Biobank recruitment), multiple socio-economic deprivation scores (Townsend deprivation index, income score, employment score, health score, education score, housing score, and crime score; continuous linear, collected at the UK Biobank recruitment), education levels (categorial, collected at the UK Biobank recruitment), body mass index (continuous linear, collected at the UK Biobank recruitment), a list of pre-specified medications (binary, obtained from primary care prescription records), the number of hospital admissions (continuous linear, obtained from HES), and comorbidities (binary, obtained from primary care diagnosis records). The weights for each participant were then computed based on the Rosenbaum formula: $Wi = \frac{Zi}{ei} + \frac{(1-Zi)}{(1-ei)}$.[27] To reduce unstable estimate due to extreme weights in the tails of the propensity score distribution, we used asymmetric trimming to exclude people whose propensity score was below the 1th percentile of the propensity score of the BNT162b2cohort and above the 99th percentile of the propensity score of the ChAdOx1 cohort.[28]
