# Supplementary material for: Comparative effectiveness of the BNT162b2 and ChAdOx1 vaccines against Covid-19 in people over 50
Source: Nat Commun. 2022 Mar 21;13:1519. doi: 10.1038/s41467-022-29159-x (PMC8938429; doi:10.1038/s41467-022-29159-x)
Supplement: Supplementary file 2 — Reporting Summary [file 41467_2022_29159_MOESM2_ESM.pdf]

nature portfolio

Corresponding author(s): Dani Prieto-Alhambra

Last updated by author(s): Jan 31, 2022

Reporting Summary

Nature Portfolio wishes to improve the reproducibility of the work that we publish. This form provides structure for consistency and transparency in reporting. For further information on Nature Portfolio policies, see our Editorial Policies and the Editorial Policy Checklist.

Statistics

For all statistical analyses, confirm that the following items are present in the figure legend, table legend, main text, or Methods section.

n/a	Confirmed	
[ ]	[x]	The exact sample size (*n*) for each experimental group/condition, given as a discrete number and unit of measurement
[x]	[ ]	A statement on whether measurements were taken from distinct samples or whether the same sample was measured repeatedly
[ ]	[x]	The statistical test(s) used AND whether they are one- or two-sided<br>*Only common tests should be described solely by name; describe more complex techniques in the Methods section.*
[ ]	[x]	A description of all covariates tested
[x]	[ ]	A description of any assumptions or corrections, such as tests of normality and adjustment for multiple comparisons
[ ]	[x]	A full description of the statistical parameters including central tendency (e.g. means) or other basic estimates (e.g. regression coefficient) AND variation (e.g. standard deviation) or associated estimates of uncertainty (e.g. confidence intervals)
[x]	[ ]	For null hypothesis testing, the test statistic (e.g. *F*, *t*, *r*) with confidence intervals, effect sizes, degrees of freedom and *P* value noted<br>*Give P values as exact values whenever suitable.*
[x]	[ ]	For Bayesian analysis, information on the choice of priors and Markov chain Monte Carlo settings
[x]	[ ]	For hierarchical and complex designs, identification of the appropriate level for tests and full reporting of outcomes
[ ]	[x]	Estimates of effect sizes (e.g. Cohen's *d*, Pearson's *r*), indicating how they were calculated

Our web collection on statistics for biologists contains articles on many of the points above.

Software and code

Policy information about availability of computer code

Data collection: No software used for data collection

Data analysis:
All data management and analyses were carried out in R (version 4.0.4).
RStudio version 2021.09.1
R package: "tidyverse" 1.3.1
R package: "survival" 3.2-13
R package: "survminer" 0.4.9
R package: "MatchIt" 4.3.2
R package: "Matching" 4.9-11

For manuscripts utilizing custom algorithms or software that are central to the research but not yet described in published literature, software must be made available to editors and reviewers. We strongly encourage code deposition in a community repository (e.g. GitHub). See the Nature Portfolio guidelines for submitting code & software for further information.

Data

Policy information about availability of data

All manuscripts must include a data availability statement. This statement should provide the following information, where applicable:

- Accession codes, unique identifiers, or web links for publicly available datasets
- A description of any restrictions on data availability
- For clinical datasets or third party data, please ensure that the statement adheres to our policy

The data used in the present study is available from UK Biobank with restrictions applied. Data was used under license, and thus not publicly available. Bonafide

researchers can apply to use the UK Biobank dataset by registering and applying at http://ukbiobank.ac.uk/register-apply/. The additional summary datasets generated and/or analysed in the current study are provided in the supplementary file.

Field-specific reporting

Please select the one below that is the best fit for your research. If you are not sure, read the appropriate sections before making your selection.

- [x] Life sciences
- [ ] Behavioural & social sciences
- [ ] Ecological, evolutionary & environmental sciences

For a reference copy of the document with all sections, see nature.com/documents/nr-reporting-summary-flat.pdf

Life sciences study design

All studies must disclose on these points even when the disclosure is negative.

Sample size	We calculated the minimal sample size based on a cohort design, where the expected incidence for the primary outcome (Covid-19 infection) in reference group was ~0.002. With the 95% confidence level and 80% power, a total of 78286 (39143 in each group) can detect a relative risk >= 1.5 or <=0.75.
Data exclusions	Scotland and Wales participants were excluded due to lack of linkage to the primary care data source where the Covid-19 vaccination information were routinely collected. Part of participants from England were also excluded due to missed data on vaccination types/brands. See the manuscript for details.
Replication	All analyses and results could be replicated for Bonafide researchers with access to UK Biobank dataset. Specifically, we included 235,181 UK Biobank participants aged 50 years or older and vaccinated with one or two doses of BNT162b2 or ChAdOx1 from 2020-12-01 to 2021-10-18. Details can be found in the Method section in the main text. Replication can be fully replicated with the same selected cohort.
Randomization	Propensity score inverse probability weighting was used to mimic randomization.
Blinding	This is an observational study with vaccination data collected routinely in clinical practices. All participants were informed by their doctor's about the vaccine types received, therefore, blinding is not possible.

Reporting for specific materials, systems and methods

We require information from authors about some types of materials, experimental systems and methods used in many studies. Here, indicate whether each material, system or method listed is relevant to your study. If you are not sure if a list item applies to your research, read the appropriate section before selecting a response.

Materials & experimental systems

n/a	Involved in the study	
x		Antibodies
x		Eukaryotic cell lines
x		Palaeontology and archaeology
x		Animals and other organisms
	x	Human research participants
x		Clinical data
x		Dual use research of concern

Methods

n/a	Involved in the study	
x		ChIP-seq
x		Flow cytometry
x		MRI-based neuroimaging

Human research participants

Policy information about studies involving human research participants

Population characteristics	The UK Biobank recruited more than 500,000 participants aged 40–69 years between 2006 and 2010. The final eligible cohort had mean age of 71 years old, 44% of male and 91% of White ethnicity.
Recruitment	Participants visited 1 of 22 assessment centers in England,Wales, and Scotland, where they completed a self reported touchscreen questionnaire; underwent physical measurements; and provided biological samples that were used for various types of arrays.
Ethics oversight	The initial protocol of the UKBB study is available (https://www.ukbiobank.ac.uk/media/gnkeyh2q/study-rationale.pdf), which has ethical approval from its own Ethics Advisory Committee (EAC) (https://www.ukbiobank.ac.uk/ethics/). The participants gave informed consent and public involvement are detailed online (https://www.ukbiobank.ac.uk/learn-more-about-uk-biobank). This project was granted under the application of 65397.

Note that full information on the approval of the study protocol must also be provided in the manuscript.
